# Results after introduction of a hip fracture care pathway: comparison with usual care

**DOI:** 10.1080/17453674.2019.1710804

**Published:** 2020-01-13

**Authors:** Stian Svenøy, Leiv Otto Watne, Ingvild Hestnes, Marianne Westberg, Jan Erik Madsen, Frede Frihagen

**Affiliations:** a Division of Orthopaedic Surgery, Oslo University Hospital;; b Institute of Clinical Medicine, University of Oslo;; c Department of Geriatric Medicine, Oslo University Hospital, Norway

## Abstract

Background and purpose — We established a care pathway for hip fracture patients, a “Hip Fracture Unit” (HFU), aiming to provide better in-hospital care and thus improve outcome. We compared the results after introduction of the HFU with a historical control group.

Patients and methods — The HFU consisted of a series of measures within the orthopedic ward, such as reducing preoperative waiting time, increased use of nerve blocks, early mobilization, and osteoporosis treatment. 276 patients admitted from May 2014 to May 2015 constituted the HFU group and 167 patients admitted from September 2009 to January 2012 constituted the historical control group. Patients were followed prospectively up to 12 months post fracture.

Results — Mean preoperative waiting time was 24 hours in the HFU group and 29 hours in the control group (p = 0.003). 123 patients (47%) in the HFU were started on anti-osteoporosis treatment while in hospital. “Short Physical Performance Battery” score (SPPB) was mean 5.5 in the HFU group and 3.8 in the control group at 4 months (p < 0.001), and 5.7 vs. 3.6 at 12 months (p < 0.001). The mortality rate at 4 months was 15% in both groups. No statistically significant differences were found in readmissions, complications, new nursing home admissions, in Barthel ADL index or a mental capacity test at the follow-ups.

Interpretation — We found improved preoperative waiting time and better SPPB score at 4 and 12 months postoperatively after introducing the HFU.

## 

Elderly hip fracture patients often suffer from comorbidities and the risk for complications is substantial (Haentjens et al. 2010, Smith et al. 2014, Ali and Gibbons 2017). Complication rates are known to increase with prolonged preoperative waiting time (Simunovic et al. 2010, Westberg et al. 2013, Pincus et al. 2017) and acceptable waiting times according to guidelines and national recommendations vary from 24 to 48 hours (AAOS 2014, NICE 2017). The recovery phase after surgery also inflicts a variety of challenges. Loss of function and independence is a risk. About half the hip fracture patients may not regain their pre-fracture mobility level and ability to perform daily activities, which may lead to loss of independence and result in transfer into a permanent care facility (Prestmo et al. 2015, Dyer et al. 2016).

Improved perioperative care and early rehabilitation may reduce mortality, prevent loss of function, and be cost effective (Kristensen et al. 2016, NICE 2017). Comprehensive geriatric assessment and orthogeriatrics are recommended for hip fracture patients (Grigoryan et al. 2014, Wang et al. 2015, Eamer et al. 2018, Nordstrom et al. 2018). Quality improvement may also be possible using existing resources within an orthopedic department and without orthogeriatrics, but less has been written about such endeavors (Larsson et al. 2016, Haugan et al. 2017). To our knowledge, no intervention study to date has shown improved functional outcome by introducing a hip fracture care program without formal collaboration with geriatricians (Panella et al. 2018). We established a care pathway, the “Hip Fracture Unit” (HFU), in our department in May 2014, relying mainly on internal resources from the orthopedic department and without orthogeriatric intervention. The HFU was constituted of elements thought to improve the quality of care, such as reducing preoperative waiting time, preoperative femoral nerve block to reduce opiates, early mobilization, and secondary prophylaxis (Lyles et al. 2007, AAOS 2014, Kristensen et al. 2016, NICE 2017, Pincus et al. 2017, Aprato et al. 2018, White et al. 2018). The main aim of this study was to find out whether implementation of an HFU was associated with improved outcome.

## Patients and methods

We carried out a single-center cohort study with historical controls. In both groups, the patients were prospectively registered. The patients in the HFU group were included from May 2014 to May 2015 and the patients in the control group were included from September 2009 to January 2012.

### Organization of the Hip Fracture Unit

The HFU was established to (a) improve preoperative routines to get the patients to surgery faster, and (b) improve postoperative treatment and routines by increasing focus on early rehabilitation, preventing complications, and enhancing secondary prevention. Interdisciplinary groups were established with orthopedic surgeons, geriatricians, anesthetists, physiotherapists, and orthopedic nurses to outline the clinical routines. Checklists for nurses working on the ward were developed. A “hip fracture nurse” was in charge of the introduction of the care pathway routines in the ward. She was also responsible for educating the other nurses and worked on adherence to routines throughout the intervention, supervised by a senior orthopedic surgeon. Liaisons with pre-hospital services, emergency ward, radiology department, hospital orderlies, clinical biochemistry, and the department of anesthesia were established. A “fast track” admission was planned with direct admission to the ward via radiology, bypassing the emergency room, after conference between pre-hospital services and an orthopedic ward nurse. Regardless of whether or not the fast-track admission route was followed, prompt examination and clearance for surgery was emphasized to all personnel involved, including anesthetists and senior trauma surgeons responsible for emergency surgery prioritizing. A preoperative evaluation by an anesthetist was formalized. Physiotherapists were present in the ward every day including weekend and holidays, to ensure patient mobilization and training (Table 1). Geriatricians, nutritionists, and occupational therapists were not part of the HFU due to lack of availability. We established a separate admission room on the ward for hip fracture patients. Access to fast track admission was 8 a.m.–8 p.m. on weekdays, and was the preferred route of admission for all hip fracture patients during opening hours when capacity allowed.

**Table 1.** Ward description

Description of the orthopedic ward n	
Number of beds	52
Staff order, numbers per bed	
Nurses	1.2
Nursing assistants	0.06
Physiotherapists	0.07
Occupational therapists	0
Nutritionists	0
Social worker	0.02

Preoperative assessment included early examination by the orthopedic surgeon on call and the anesthetist, including nerve block to reduce opioid use (Table 5). The hip fracture nurse registered when nerve block was administered. No changes were made in surgical methods guidelines. Patients were aimed to be mobilized on the first postoperative day and the hip fracture nurse registered whether mobilization actually occurred. All patients were given a standard nutritional supplement drink containing a high-energy triglyceride fat emulsion, proteins, carbohydrates, and vitamins. Recommended osteoporosis treatment was cholecalciferol 100,000 IU orally and zoledronate 5 mg intravenously. When contraindications for bisphosphonates were present, the patients were started on denosumab 60 mg subcutaneously. Patients were also given a daily oral supplement with calcium (500–1,000 mg) and vitamin D (800 IU). Falls prevention was assessed individually.

**Table 5.** Results for the Hip Fracture Unit (HFU) during hospital stay. Values are frequency (%) unless otherwise specified

Variable	Aim (%)	Results
Number of patients	–	276
Preoperative femoral nerve block	–	155/260 (60) **^a^**
Mean (SD) preoperative waiting time, h	–	24 (12)
Operated within 48 h	(100)	260/271 (96) **^a^**
Operated within 24 h	(> 80)	157/272 (58) **^a^**
Mobilization started within first day after surgery	(100)	220/240 (92) **^a^**
Urethral catheter removed before 36 hours post-surgery	(> 80)	147/250 (59) **^a^**
Started anti-osteoporosis drug treatment during stay^b^	(> 80)	123/264 (47)^a^
Admitted to other orthopedic wards than the HFU ward	None	62/276 (22)
Duration of hospital stay, median days (range)	5–7	5 (2–30)

a Total number reduced due to missing data.

b For anti-osteoporosis drugs used, see Table 3

The HFU in the orthopedic ward was established on May 7, 2014, and patients were included for the present study during the first 12 months. Patients with high-energy trauma and patients living in other hospital regions were excluded from the present analyses (Figure 1).

**Figure:**
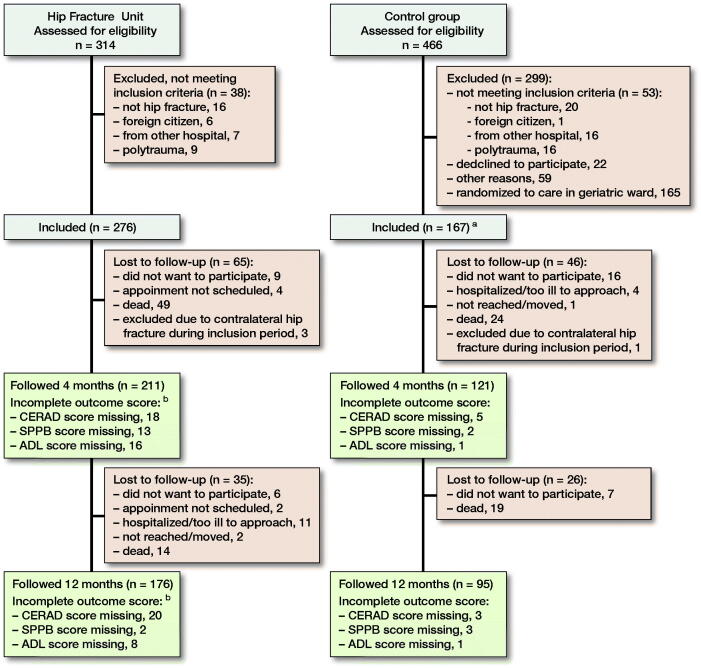
Patient inclusion and follow-up. **^a^**Randomized to “usual care” in the orthopedic ward in original study (165 patients were randomized to admission in the geriatric ward and are not included in the present study). **^b^**In addition, 11 patients at 4 months and 5 patients at 12 months are missing scores due to surgeons other than SS and FF seeing the patients.

#### Historical control group

Data from 167 patients from a previous trial in our hospital on hip fractures, the Oslo Orthogeriatric Trial (OOT), constituted the historical control group (Watne et al. 2014). These 167 patients were the group randomized to “usual care”, i.e., admission to the orthopedic ward.

#### Outcome measures

The outcome measures for comparisons with the historical control group were taken from post-discharge outcomes from the OOT (Watne et al. 2014). In addition, selected quality indicators were chosen to evaluate the performance of the HFU (Tables 3 and 5).

**Table 3.** Results and complications before and after introduction of the Hip Fracture Unit

Variable	HFU group n = 276	Control group n = 167	Relative risk and/or (CI)
Waiting time before surgery, mean h	24	29	(1.6–7.6)
Operated within 48 hours, n (%)	260/271 (96)	139/163 (85)	1.1 (1.0–1.2)
Operated within 24 hours, n (%)	157/271 (58)	83/163 (51)	1.1 (0.9–1.4)
Started anti-osteoporosis drug			
treatment during stay, n (%)	123/273 (47)	1 (0.5)	
Zoledronic acid	95	1	
Alendronate	3	0	
Denosumab	25	0	
Days of hospital stay, median (range)	5 (2–30)	8 (1–39)	(2.2–3.7)
Postoperative surgical complications, n (%)	22 (8)	8 (5)	1.6 (0.7–3.6)
Deep wound infection	9	1	
Prosthetic dislocation	2	3	
Fixation failure	6	3	
Other **^a^**	5	1	
Readmitted in 30 days, n (%)	32/271 (12)	13/165 (8)	1.5 (0.8–2.7)
Reoperated at 4 months, n (%)	16/276 (6)	5/164 (3)	1.9 (0.7–5.1)
at 12 months (accumulated), n (%)	18/276 (7)	9/164 (6)	1.2 (0.5–2.5)
New admissions to a permanent care			
facility at 4 months, n (%)^b^	41/172 (24)	18/83 (22)	1.1 (0.6–1.7)
Dead at 4 months	43 (15)	25 (15)	1.0 (0.6–1.6)
Dead at 12 months	63 (23)	43 (26)	0.9 (0.9–1.1)

a Other surgical complications were: 1 trochanteric avulsion. 1 peroneal nerve palsy. 1 reoperated for hematoma. 1 hemiarthroplasty had soft tissue interposed in the acetabulum. 1 reoperated for gluteus medius insufficiency. 1 had a skin laceration of the leg after a fall in the ward.

b Patients who lived in a permanent care facility preoperatively were excluded.

Both the patients from the HFU and the patients in the control group were followed up at 4 and 12 months postoperatively. The HFU patients were seen in the outpatient clinic by an orthopedic surgeon and the control group were seen on home visits by a trained study nurse. The tests used were: the Consortium to Establish a Registry for Alzheimer’s disease (CERAD), used to measure cognitive function, where patients were asked to recall a set of 10 words presented to them visually (Welsh et al. 1994); the Short Physical Performance Battery (SPPB), combining results of gait speed, balance, and repeated chair stands, a score for mobility and function (Guralnik et al. 1994); the Barthel activities of daily life (ADL) index was used to indicate the patients’ degree of independence (Mahoney and Barthel 1965).

Other outcomes were surgical complications, readmissions, reoperations, secondary prophylaxis, and mortality. The outcomes were registered during the hospital stay and throughout the follow-up period.

### Statistics

No formal power calculation was performed as the number of patients available for the historical control groups was fixed. The number of patients included from the HFU was decided based on the power calculations in the OOT using a composite cognitive function outcome measure as primary outcome measure (Watne et al. 2014) and the other Norwegian RCT on orthogeriatrics using the SPPB (Prestmo et al. 2015).

Normality tests including Q–Q plots and the Kolmogorov–Smirnov test were used for evaluating data distribution. Variables were analyzed by independent sample t-test, Mann–Whitney U-test and chi-square test depending on the data distribution. Some of the variables were not normally distributed. In these cases, non-parametric tests (Mann–Whitney U) were also performed as sensitivity analyses producing basically the same results. Fisher’s exact test was used where some of the cell numbers were low. Multiple linear regression analyses were performed to investigate the relationships between the HFU and the control group on functional outcome; CERAD, SPPB, and ADL scores were chosen as dependent variables, and variables believed to influence the outcome (sex, age, ASA score, and pre-fracture residency) were chosen as independent variables. A p-value of < 0.05 was considered statistically significant. Data are presented with percentages, relative risks (RR), and 95% confidence intervals (CI) were appropriate. We used SPSS for Windows version 24 (IBM Corp, Armonk, NY, USA).

#### Ethics, registration, funding, and potential conflicts of interest

The OOT was approved by the regional ethics committee (REK 2009/450). The quality register of the HFU population was approved by the Data Protection Officer (2014/12309 and 2014/1433 REK). The work was funded by the hospital. The authors declare no conflict of interest.

## Results

From May 2014 to May 2015, 314 patients were assessed for eligibility for the HFU, and 276 patients were included (Figure 1). There were more women in the historical control group and a larger proportion living in an institution before the injury (Table 2).

**Table 2.** Baseline characteristics of the Hip Fracture Unit (HFU) care pathway and the historical control group. Values are frequency (%) unless otherwise specified

Baseline characteristics	HFU intervention group n = 276	OOT **^a^**control group n = 167
Female sex	185 (67)	129 (77)
Age^b^	81 (11) [80–82]	82 (10) [81–84]
median (range)	84 (49–98)	85 (46–101)
ASA^b^	2.6 (0.7) [2.5–2.7]	2.6 (0.6) [2.5–2.7]
Living in institution	59 (21)	51 (30)
Type of fracture		
Femoral neck	151 (56)	98 (59)
Trochanteric	113 (42)	67 (40)
Subtrochanteric	5 (2)	2 (1)
Surgical procedure		
Arthroplasty	127 (46)	72 (43)
Osteosynthesis	149 (54)	91 (55)
Not operated	0 (0)	4 (2)
Duration of surgery, min^b^	71 (31) [67–74]	77 (43) [71–84]

a Oslo Orthogeriatric Trial.

b Values are mean (SD) [95% CI]

### 

#### Comparison of the HFU group with the historical control group

332 patients were assessed at 4 months’ follow-up (211 patients in the HFU group and 121 patients in the control group). 62 patients were lost to follow-up at 12 months (Figure 1). There were no obvious differences between those lost to follow-up and those who were followed by sex (47/62 [76%] females versus 200/271 [74%] females), age (mean 80 years versus mean 81 years), or ASA score (mean 2.5 versus 2.4).

Mean preoperative waiting time from admission to surgery start was reduced by 4.6 hours after introduction of the HFU (Table 3). The HFU group had better SPPB scores at 4 and 12 months than the control group by about 2 points (Table 4). The improvement in SPPB scores was still present after the regression analysis (Table 4). The groups had similar results regarding CERAD and ADL in the adjusted analyses.

**Table 4.** Functional outcome at 4 and 12 months postoperatively. Values are mean (SD)

Variable **^a^**	HFU group	Control group	(CI)	Adjusted analysis^b^	
				p-value	B (CI)
4 months results					
SPPB	5.5 (4.7)	3.8 (3.4)	(0.7 to 2.6)	0.03	0.9 (0.1 to 1.7)
CERAD	13.3 (7.6)	11.4 (7.5)	(0.1 to 3.7)	0.5	0.5 (–0.9 to 1.9)
Barthel ADL	15.7 (5.2)	14.6 (5.5)	(–0.1 to 2.2)	0.8	–0.1 (–1.1 to 0.9
12 months results					
SPPB	5.7 (4.7)	3.6 (3.3)	(0.5 to 1.1)	0.02	1.0 (0.2 to 2.0)
CERAD	14.2 (8.3)	11.5 (8.4)	(0.4 to 4.8)	0.8	0.2 (–1.5 to 1.8)
Barthel ADL	16.6 (4.8)	14.3 (5.6)	(0.9 to 3.5)	0.1	0.9 (–0.1 to 2.0)

a Short Physical Performance Battery (SPPB) scale 0–12, Consortium to Establish a Registry for Alzheimer’s disease (CERAD) scale 0–30, Barthel Activities of Daily Life (ADL) scale 0–20.

b Linear regression on mean difference adjusted for age, gender, ASA, and pre-fracture accommodation (living in institution or home-dwelling).

There was no improvement in readmissions, complications, or mortality after introduction of the HFU.

#### Fast track admission in the Hip Fracture Unit

260/271 (96%) of the patients were operated within 48 hours after admission (Table 5). 55/276 (20%) patients were admitted through the fast-track pathway. 129/276 patients (47%) were admitted during fast-track opening hours, but 74 of those 129 (57%) were still not admitted through the fast track. The reasons were: failed to alert fast-track team prehospital for 35 patients, lack of ward nurse capacity for 36 patients, and 3 patients were deemed medically unfit for direct ward admission.

## Discussion

We found improved functional outcome at 4 and 12 months postoperatively in the HFU group measured by SPPB compared with the historical controls (see Table 4). We improved several key areas of care compared with the historical control group, including preoperative waiting time and osteoporosis treatment (Table 3). We had, however, low performance in some other areas; perhaps most notably that only 1 in 5 patients were admitted through the fast-track pathway. There was no improvement in readmissions or new nursing home admissions. An increased number (p = 0.2) of surgical complications was seen in the HFU group) (Table 3).

The relationship between preoperative delay and increased morbidity and mortality is well published and has led to guidelines recommending surgery within 24–48 hours (AAOS 2014, NICE 2017). A retrospective study found increased 30-day mortality and more medical complications when preoperative waiting time exceeded 24 hours (Pincus et al. 2017).

To our knowledge, no intervention to reduce preoperative delay showing improved outcome on function, morbidity, and mortality has been published. A randomized controlled trial on fast-track admission versus traditional care pathway in 571 patients found essentially no differences between the groups (Larsson et al. 2016). In the retrospective study from Haugan et al. (2017), admission and ward routines were organized as an HFU similar to ours. They found decreased time to surgery and decreased length of stay (LOS) with fast-track admission compared with usual care. Nevertheless, no differences were observed in mortality or readmission rate. A multicenter quality improvement study from 2018, comparing a care pathway with usual admission and care, found no differences in mortality or functional outcome (Panella et al. 2018). An RCT on preoperative waiting time is being conducted and the results from that trial will probably elucidate this question (Borges et al. 2019). Reduced preoperative waiting time may reduce the LOS, but we believe that the reduced LOS in the HFU was mainly due to improved capacity in the municipal health services (Table 3) (Monkerud and Tjerbo 2016).

The SPPB was used to evaluate postoperative function in the former RCT constituting our historical control group (Watne et al. 2014). The minimally meaningful change in SPPB score has been estimated to be 0.5 units (Perera et al. 2006). The between-group difference in our study suggest a clinically meaningful difference (see Table 4). In contrast, the Barthel ADL index showed no statistically significant improvement. There was a concern regarding a high number of postoperative complications in the HFU (8% vs. 5%). This was mainly driven by the difference in deep wound infections. The numbers were low and the difference was not statistically significant (p = 0.2). An increased number of complications may be an effect of 1 or more components of our HFU, but we have failed to find an obvious link. In previous publications from our department the rate of postoperative deep infections varied from 1% to 9%, still with no apparent explanation for the differences (Frihagen et al. 2007, Westberg et al. 2013, Guren et al. 2017).

The intervention in the HFU consisted of multiple smaller elements thought to improve care. It is not possible to discern which elements were beneficial, indifferent, or even harmful for the patients with hip fractures. Adherence to routines varied even though quality improvement work was done by the “hip fracture nurse” and the lead surgeon throughout the study period. None of our predefined aims for quality of care were completely reached after establishing the HFU (Table 5).

### 

#### Orthogeriatric care

As described by Haugan et al. (2017), the organization of a clinical care pathway is based on principles from lean methodology. The key concept is standardization of routines and reducing variation in treatment (Niemeijer et al. 2013). Comprehensive geriatric assessment is established in the treatment for hip fractures in some countries, and meta-analyses of high-quality trials have reported improved outcome (Grigoryan et al. 2014, Wang et al. 2015, Eamer et al. 2018, Nordstrom et al. 2018). Introducing orthogeriatric care may, however, be difficult both due to financial and logistical issues, and to shortage of geriatricians. The formation of our HFU was inspired by improvements shown by introducing orthogeriatric care, but due to restraint on resources and logistics we were not able to establish an orthogeriatric service (Grigoryan et al. 2014, Prestmo et al. 2015, Eamer et al. 2017, Eamer et al. 2018, Nordstrom et al. 2018). Our perspective may be relevant in healthcare systems where geriatric resources are unavailable or where there is lack of willingness to pay for interdisciplinary services. Our HFU also had elements of a fast-track pathway, but the low number of patients using this pathway may imply that our HFU did not have adequate resources or a robust enough solution for the admission routine. Even with limited “opening hours” due to ward staffing we still had problems maintaining the admissions routine when it should have been available. From this experience we will in the future seek to establish routines that are intended to be available 24/7.

#### Strength and weaknesses of the study

The correlation between improvements made by introducing the HFU and the clinical scores at 4 and 12 months requires careful interpretation. Patients in the HFU were about 1.5 years younger and 9% fewer patients lived in an institution preoperatively. On the other hand, ASA scores for the 2 groups were similar. There were also more men in the HFU group, and studies have indicated that being male increases the risk of mortality and adverse events after a hip fracture (Bretherton and Parker 2015, Sathiyakumar et al. 2015). Still, there may have been systematic differences between the groups that our regression analysis failed to adjust for. Papers evaluating a quality improvement effort like ours are vulnerable to misinterpreting multifactorial improvements over time as being a result of the intervention, so any positive finding should be interpreted with caution.

The patient populations were included in 2 different time periods and an improvement of care may have come about independently of our intervention, through an increased awareness of the needs of hip fracture patients.

The functional outcome data were collected at home visits for the control group and at outpatient clinic visits for the HFU group. The evaluators performing the home visits in the control group were blinded to treatment group, but the orthopedic surgeons examining the HFU group were part of the HFU team. This may have led to bias. It does not, however, seem that it led to a selection of healthier patients in the HFU follow-ups, and there were still fewer patients lost to follow-up in the HFU group, although more patients had missing individual outcome scores. Some patients were mistakenly scheduled for follow-up appointment with other colleagues who were not aware of the study follow-up protocol. Thus, the clinical scores were not obtained for these patients (Figure 1).

Our control group was part of an earlier RCT with wide inclusion criteria, and we believe it to be close to an unbiased population. The intervention group was also unselected, and loss to follow-up was low in both groups. The main strengths of our study are the prospective registration and the systematic follow-up, including functional outcome measures.

## Conclusion

A low-cost HFU within the orthopedic department may be a tempting alternative when resources are limited. We failed in our attempt at fast-track admission, but reduced mean time to surgery by almost 5 hours and achieved reasonable numbers on our performance indicators. Apart from the improved SPPB during follow-up, we found limited or no effect of our HFU after discharge. A care pathway like ours may still be attempted in hospitals where quality improvement is sought. In our experience, however, some resources must be added to initiate and sustain a care pathway, and the literature supports the addition of comprehensive geriatric assessment and the formation of a strong interdisciplinary team.

All the authors were responsible for planning the study. SS performed the statistical analyses and wrote the first draft. All authors participated in the interpretation of data, and critical revision of the manuscript. We thank research nurses Elisabeth Fragaat, Tone Fredriksen, Camilla Marie Andersen, Julie Ask Ottesen, Linda Feldt, Sissel Knuts, and Elise Berg Vesterhus for assisting in data collection.

*Acta* thanks Pia Kjaer Kristensen for help with peer review of this study.
